# Successful Management of Angiostrongylus Cantonensis-Induced Eosinophilic Meningitis Using Albendazole–Corticosteroid Therapy: A Case Report With Serial Cytokine and CSF Monitoring

**DOI:** 10.1155/crdi/7526279

**Published:** 2025-10-09

**Authors:** Wen-Dong Cong, Min Yu, Si-Man Chen, Peng-Fei Qin, Qing-Mei Huang, Zhan Gao, De-Feng Liu, De-Tian Liu

**Affiliations:** ^1^Department of Neurology, Longgang Central Hospital of Shenzhen, Shenzhen, Guangdong, China; ^2^Department of Clinical Laboratory, Longgang Central Hospital of Shenzhen, Shenzhen, Guangdong, China; ^3^Department of Anesthesiology, Traditional Chinese Medicine Hospital of Zijin County, Heyuan, Guangdong, China

**Keywords:** albendazole, *Angiostrongylus cantonensis*, clinical case, eosinophilic meningitis, inflammatory cytokines

## Abstract

Angiostrongylus eosinophilic meningitis is aparasitic disease caused by *Angiostrongylus cantonensis*. The initial report is originated from southern China. However, the incidence rate has decreased due to improvements in sanitary conditions. Individuals who become infected are considered accidental cases. The clinical symptoms exhibit variability, with eosinophilic meningitis accompanied by elevated intracranial pressure being the most prevalent clinical presentation. The main treatment for angiostrongylus eosinophilic meningitis involves giving a combination of albendazole and corticosteroids. In this report, we present a prototypical case of eosinophilic meningitis and confirm *Angiostrongylus cantonensis* as the causative pathogen through next-generation sequencing (NGS). The disease progression was monitored using a range of blood and cerebrospinal fluid (CSF) assays. The patient underwent an extensive 46-day systemic treatment, resulting in a remarkable reduction of inflammatory cytokines and eosinophilic cells. The combined therapy of albendazole and corticosteroids, along with dehydration management and neuroprotective measures, resulted in positive outcomes. The timely detection and prompt initiation of anthelmintic therapy are associated with a favorable prognosis. This successful experience provides valuable insights for clinical practice.

## 1. Introduction

Neuroangiostrongyliasis is a zoonotic parasitic disease caused by the infection of *Angiostrongylus cantonensis*. Clinical cases of the disease clearly demonstrate two aspects: regional-related, with the majority of cases from southern China or other parts of Southeast Asia, and dietary-related, where raw or slightly cooked snails are regarded as a delicacy [1, 2]. *Angiostrongylus cantonensis* was presumably first discovered in southern China in 1933 [3–5]. However, this parasitic disease has become globally transmitted in tropical regions [3]. By 2017, there had been approximately 3000 reported cases worldwide [4, 5]. Infected humans were accidental hosts, mostly infected by consuming raw or undercooked snails, fish, and shrimp that contained third-stage larvae [6]. They were also infected through the consumption of vegetables, fruits, and drinking water contaminated with third-stage larvae [2]. The larvae of *Angiostrongylus cantonensis* enter the human digestive tract through the oral cavity, penetrate the intestinal wall, and subsequently migrate to the brain via the circulatory system by traversing the blood–brain barrier, ultimately invading the central nervous system, with a higher prevalence in the cerebellum followed by the diencephalon and brainstem [7, 8]. The neurological manifestations encompass eosinophilic meningitis, encephalitis/encephalomyelitis, radiculitis, cranial nerve abnormalities, ataxia, or paralysis [8]. Additionally, some patients may exhibit hepatomegaly, ocular lesions, pulmonary hemorrhage, granulomatous pneumonia, and other related symptoms [9]. Among these symptoms, the most commonly observed clinical presentation is eosinophilic meningitis accompanied by elevated intracranial pressure [10]. The incubation period for eosinophilic meningitis typically lasts around 14 days, which aligns with the duration required for the migration of third-stage larvae into central nervous system [10]. The routine blood tests revealed varying degrees of increased eosinophils counts. The cerebrospinal fluid (CSF) exhibits inflammatory alterations, concomitant with intracranial hypertension [9]. The number of white blood cells in the CSF gradually increases with the duration of the disease, and the count of white blood cells can reach 500–2000∗10^6^/L, primarily attributed to an elevation in eosinophils [11, 12]. The enzyme-linked immunosorbent assay (ELISA) is one of the most commonly used methods for diagnosing the disease [13]. Also, it can be rapidly and accurately detected in CSF using next-generation sequencing (NGS) technology [11]. Currently, there are no consensus pharmacological interventions available for the treatment of angiostrongyliasis [14]. The primary approach consists of anthelmintic treatment, symptomatic, and supportive therapies. The clinical effectiveness and safety of albendazole in the treatment of neuroangiostrongyliasis have been reported [14]. The use of albendazole–corticosteroid therapy is recommended due to the potential inflammation caused by the killed larvae and associated complications [7]. However, this treatment course remains controversial because combination therapy has not been tested in a double-blind study [8], and corticosteroids seem to be ineffective in severe eosinophilic meningitis [15]. In this study, we present a case that underwent two courses of albendazole–corticosteroid therapy. We sincerely hope that the experience of this successful case can be disseminated.

## 2. Case Presentation

### 2.1. Chief Complaint and Clinical Presentation

We present a case of a 36-year-old male patient (weight: 60 kg) who was in good health, with no history of chronic diseases or medication use. The patient showed symptoms of headache and generalized myalgia (Numeric Rating Scale: 8/10), a low-grade fever with temperature fluctuations ranging between 37 and 38 degrees Celsius, and a state of apathy.

### 2.2. Exposure

The patient ate some Chinese River Snails (*Cipangopaludina chinensis*) at a barbecue restaurant twenty days ago before being taken to the hospital. The presence of other snail species, including Apple Snail (*Pomacea canaliculata*), in this batch of field snails remains unverified. The Chinese River snails were prepared by stir-frying, which is a prevalent culinary practice among local residents.

### 2.3. Physical Examination

Nervous system examination revealed that the patient was lethargic and confused, had difficulty understanding speech, and had positive nuchal rigidity, and a positive Kernig's sign.

### 2.4. Laboratory and Imaging Findings

**Figure 1:**
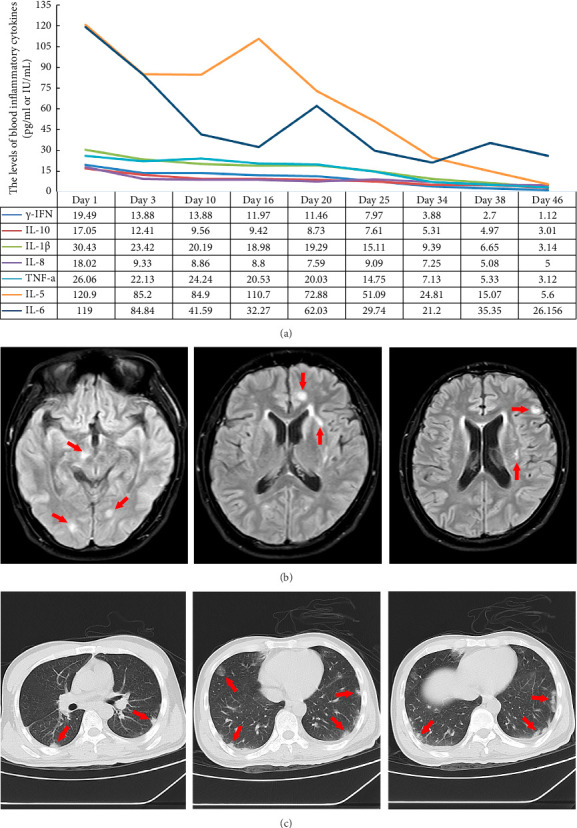
(a) The levels of inflammatory cytokines (γ-IFN, IL-10, IL-1β, IL-8, IL-5, IL-6, and TNF-α) exhibit a decreasing trend, particularly for IL-5 and IL-6. The unit of γ-IFN is international units per milliliter (IU/mL). The units of IL-10, IL-1β, IL-8, IL-5, IL-6, and TNF-α are picograms per milliliter (pg/mL). (b) The cranial magnetic resonance imaging scan showed subcortical abnormalities in both cerebral hemispheres on the T2-FLAIR sequence. (c) The lung CT scan showed several distinct inflammatory signal changes near the pleura.

**Figure 2:**
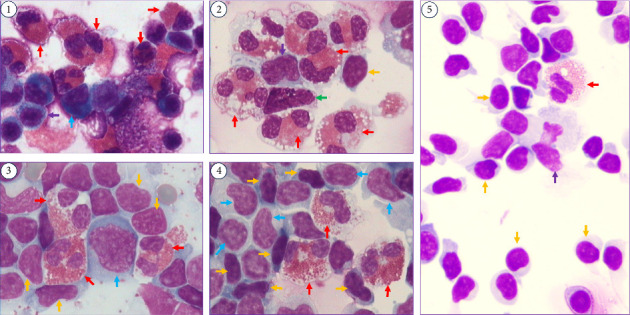
Five CSF cytology test results are presented. Red arrows indicate eosinophil cells. Yellow arrows indicate mature lymphocyte cells. Green arrows indicate basophil cells. Purple arrows indicate mononuclear macrophage cells. Azure blue arrows indicate stimulated lymphocyte cells. The ratio of eosinophilic cells in the cytology revealed a significant decrease from figure ① to ⑤. The ratio of mononuclear cells (such as mature lymphocyte cells, indicated by yellow arrows) shows an increasing tendency. The ratio of multinucleate cells (such as eosinophil cells, indicated by red arrows) shows a decreasing tendency.

The routine blood test revealed a significant elevation in eosinophilic granulocytes (Table 1). The blood inflammatory cytokines, including gamma interferon (γ-IFN), interleukin-10 (IL-10), interleukin-1β (IL-1β), interleukin-8 (IL-8), interleukin-5 (IL-5), interleukin-6 (IL-6), and tumor necrosis factor-α (TNF-α), were used to assess the disease. The expression levels of IL-5 and IL-6 exhibited significantly higher expression compared to other inflammatory cytokines (Figure 1(a)). To identify the causative agent of the intracranial infection, lumbar puncture was performed. The CSF color was yellow during the first test. The intracranial pressure was above 330 mm H_2_O (Table 1). The CSF routine examination revealed an abnormal increase in cell counts and proteins, but the glucoses were decreased. The ratio of mononuclear cells accounted for 34%, and the ratio of multinucleate cells was 66%. The CSF cytology showed a significant increase in the ratio of eosinophil cells (Figure 2-①), which accounted for 60% (Table 1). NGS was used to confirm the pathogen, and it identified 174 sequences of *Angiostrongylus cantonensis*. The ELISA revealed positive antibody (IgG) results for *Angiostrongylus cantonensis* (optical density value 1.220) and *Hydatid* (optical density value 0.285). Cranial magnetic resonance imaging revealed extensive subcortical abnormalities in both cerebral hemispheres (Figure 1(b)). The lung CT scan revealed multiple isolated inflammatory signal changes adjacent to the pleura (Figure 1(c)).

### 2.5. Diagnosis

Upon a comprehensive analysis of the abovementioned data, the patient was finally diagnosed with *Angiostrongylus cantonensis*-induced eosinophilic meningitis (AEM).

### 2.6. Treatment

The patient ate some antibiotics (cephalosporin) and antipyretics (ibuprofen sustained-release capsules), but they demonstrated limited efficacy before he was taken to the hospital. The dosage and administration details of those drugs remain unspecified because the patient exhibits a compromised mental state and cannot remember clearly. In the hospital setting, early treatment measures, including antibiotics (ceftriaxone sodium), antiviral drugs (acyclovir), antituberculosis drugs (isoniazid, rifampicin, and pyrazinamide), and dehydrating agents for reducing intracranial pressure (glycerin fructose), did not achieve the expected results. When the NGS result identified the pathogenic microorganism, albendazole was administered (20 mg/kg/day, 0.4 g, TID, po, for two weeks) to manage the disease. Corticosteroids were used to suppress inflammatory response (dexamethasone, 20 mg, QD, ivgtt, for 1 week). Additionally, treatment for other symptoms included managing dehydration and implementing neuroprotective measures. The patient's symptoms showed improvement after the administration of the medication. The CSF cytology showed no significant changes in the second tests conducted at the end of the initial treatment (Table 1 and Figure 2-②). The third CSF test showed a shift in the proportion of mononuclear cells (accounting for 90%) and multinucleate cells (accounting for 10%). The proportion of multinucleate cells decreased while that of mononuclear cells increased, indicating improvement (Table 1). The second NGS analysis detected 15,997 sequence reads of *Angiostrongylus cantonensis*. However, the albendazole treatment was discontinued, and the dexamethasone dosage was reduced to 10 mg (QD, ivgtt, for 1 week). The fourth CSF test remained abnormal (Figure 2-④), but the proportion of mononuclear and multinucleate cells changed significantly (Table 1). The proportion of eosinophilic cells ratio decreased, the ratio of mononuclear cells shows an increasing tendency, and the ratio of multinucleate cells shows a decreasing tendency (Table 1). Therefore, the second anthelmintic treatment (albendazole, 0.4 g, TID, po, for 1 week) was administered.

### 2.7. Outcome

The patient exhibited complete symptom resolution, with CSF results (Figure 2-⑤) and inflammatory cytokine levels (Figure 1) decreased and approaching the normal range by the 46th day. Although the final NGS test revealed 16,858 sequence reads of *Angiostrongylus cantonensis* (Table 1), the third deworming treatment was discontinued. Methylprednisolone (40 mg, QD, po) was administered to reduce the brain inflammatory reaction during the follow-up treatment. The dosage was gradually reduced by 5 mg per week for a total duration of eight weeks.

### 2.8. Follow-Up

The patient was followed for 1 year, and subsequent examinations showed normal results.

## 3. Discussion

In the current study, we employed NGS for the diagnosis of AEM. Serial cytokine and CSF measurements were used to monitor the disease. The albendazole–corticosteroid treatment regimen showed promising efficacy in managing this disease. Early diagnosis and timely administration of medication are crucial for the successful treatment of the disease. Herein, we present the successful treatment experience of this disease.

A number of inflammatory markers, such as γ-IFN, IL-10, IL-1β, IL-8, and TNF-α, particularly IL-5 and IL-6, exhibited elevated levels initially but gradually decreased after antiparasitic treatment (Figure 1(a)). IL-5 plays an essential role in orchestrating eosinophilic inflammation, enhancing the production of IgE, and promoting the maturation and survival of eosinophils [16]. IL-6 is to regulate the proliferation and differentiation of various cell types and enhances the activation of CD4^+^ T cells and B-cell responses, promoting the production of parasite-specific antibodies [17]. Elevated levels of IL-5 and IL-6 increase the immune response in the host organism during parasites infection [18]. Treatment with antiparasitic medication significantly decreased the levels of IL-5 and IL-6, indicating that IL-5 and IL-6 can serve as critical indicators for monitoring disease progression.

Five lumbar punctures were performed to track the changes of CSF cytology. A significant presence of eosinophils was observed in the CSF under light microscopy before treatment (Figure 2-①). The CSF cytology became normal occurred approximately 46 days posttreatment (Figure 2-⑤). The dynamic monitoring of CSF cytology proved valuable in assessing the improvement of the disease. The CSF DNA sequences remain elevated even in the presence of normal blood and CSF routine test, indicating a sustained anomaly. Since the patient's symptoms had completely subsided, we hypothesized that this might be attributed to the release of a significant amount of free DNA following the necrosis and disintegration of the worms. As a result, the brain would require an extended period for self-clearance. The time when the CSF DNA sequencing test becomes entirely normal still requires further tracking.

ELISA detected the presence of two types of parasitic antibodies (IgG), specifically those against *Angiostrongylus cantonensis* and *Hydatid*. Nevertheless, *Hydatid* was not detected in the CSF DNA sequencing. Moreover, the patient's symptoms did not match those typically associated with *Hydatid* infection, suggesting a previous infection. Thus, NGS technology holds significant value in distinguishing between newly acquired and preexisting infections and serves as a crucial diagnostic tool in clinical practice.

Previously, the evidence for albendazole's benefit in AEM was not statistically significant [19]. Recently, an *in vitro* study demonstrated that albendazole, ivermectin/moxidectin, and pyrantel pamoate show promise as potential candidates for the management and prevention of neuroangiostrongyliasis [20]. To mitigate immune reactions or intracranial hypertension resulting from parasite disintegration during deworming treatment, concurrent administration of corticosteroids can significantly alleviate treatment-related adverse effects [7]. A systematic review revealed that corticosteroid treatment reduces headache in patients with eosinophilic meningitis [21]. Further, high-dose corticosteroids (e.g., prednisolone 60 mg per day for at least 1–2 weeks) were the recommended treatment [8]. The utilization of albendazole and corticosteroids was widely prevalent in the management of *Angiostrongylus cantonensis* [22, 23]. Previous clinical reports that described the coadministration of corticosteroids along with an anthelmintic had led to positive outcomes [24, 25]. In our case, high dose and prolonged use of corticosteroids along with albendazole showed a good outcome. However, the combination of albendazole and corticosteroid therapy is still controversial [26]. We should notice that the use of steroids alone may result in disease progression, and the migration of third-stage larvae maybe more susceptible [7]. It is also crucial to emphasize that prompt ophthalmological intervention should be sought without delay before commencing anthelmintic treatment, because ocular angiostrongyliasis can still result in permanent visual impairment [27]. Therefore, the timing of corticosteroid use warrants further exploration.

Neuroangiostrongyliasis is a disease that prevails in tropical regions. The epidemic of this disease exhibits an outbreak pattern [28]. With the economic and social development in China, the improvement of living conditions, and the transformation of cooking habits, the incidence of this disease in our country has gradually decreased, showing the characteristics of sporadic epidemics. Precisely because of the low prevalence of this disease, there has been no substantial progress in its diagnosis and treatment. Few randomized controlled trial studies have been conducted on the corticosteroid treatment of eosinophilic meningitis [21, 24, 29]. However, large-scale, well-designed, randomized, blinded, placebo-controlled trials have not been designed. As a result, there is no globally unified treatment consensus. Further research on high-quality studies demands the unremitting commitment of scholars.

## Figures and Tables

**Table 1:** The CSF and blood test results.

Date	6/6	6/19	6/28	7/8	7/16	Ref
*CSF test*	①	②	③	④	⑤	
Color	Yellow	Clear	Clear	Clear	Clear	Clear
Intracranial pressure(mm H_2_O)	> 330	190	160	175	138	80–180
Pan's protein	±	±	+	+	−	−
White blood cell (∗10^6^/L)	628	131	213	383	62	0–8
Protein (g/L)	1765	1820	1602	1223	791	150–450
Glucose (mmol/L)	1.96	2.68	2.33	2.26	2.35	2.8–4.5
Chloride (mmol/L)	121.3	118	119.4	121.6	118.1	120–132
Mononuclear cell ratio	34%	41%	90%	90%	60	—
Multinucleate cell ratio	66%	59%	10%	10%	2	—
CSF eosinophilic cell ratio	60%	44%	25%	5%	0%	0
NGS sequence reads	174	—	15,997	—	16,858	0

*Blood test*	①	②	③	④	⑤	
White blood cell (∗10^9^/L)	9.65	9.58	6.91	7.65	8.22	3.5–9.5
Eosinophil cell ratio (%)	26.9	2.4	3	3.8	1	0.4–8
Eosinophils absolute value (∗10^9^/L)	2.6	0.23	0.21	0.29	0.08	0.02–0.52

Abbreviations: CSF, cerebrospinal fluid; NGS, next-generation sequencing.

## Data Availability

Anonymized data not published within this article will be made available by request from any qualified investigator.
